# Xylose reductase from *Pichia stipitis *with altered coenzyme preference improves ethanolic xylose fermentation by recombinant *Saccharomyces cerevisiae*

**DOI:** 10.1186/1754-6834-2-9

**Published:** 2009-05-05

**Authors:** Oskar Bengtsson, Bärbel Hahn-Hägerdal, Marie F Gorwa-Grauslund

**Affiliations:** 1 Department of Applied Microbiology, Lund University, SE-221 00 Lund, Sweden

## Abstract

**Background:**

Xylose reductase (XR) and xylitol dehydrogenase (XDH) from *Pichia stipitis *are the two enzymes most commonly used in recombinant *Saccharomyces cerevisiae *strains engineered for xylose utilization. The availability of NAD^+ ^for XDH is limited during anaerobic xylose fermentation because of the preference of XR for NADPH. This in turn results in xylitol formation and reduced ethanol yield. The coenzyme preference of *P. stipitis *XR was changed by site-directed mutagenesis with the aim to engineer it towards NADH-preference.

**Results:**

XR variants were evaluated in *S. cerevisiae *strains with the following genetic modifications: overexpressed native *P. stipitis *XDH, overexpressed xylulokinase, overexpressed non-oxidative pentose phosphate pathway and deleted GRE3 gene encoding an NADPH dependent aldose reductase. All overexpressed genes were chromosomally integrated to ensure stable expression. Crude extracts of four different strains overexpressing genes encoding native *P. stipitis *XR, K270M and K270R mutants, as well as *Candida parapsilosis *XR, were enzymatically characterized. The physiological effects of the mutations were investigated in anaerobic xylose fermentation. The strain overexpressing *P. stipitis *XR with the K270R mutation gave an ethanol yield of 0.39 g (g consumed sugars)^-1^, a xylitol yield of 0.05 g (g consumed xylose)^-1 ^and a xylose consumption rate of 0.28 g (g biomass)^-1 ^h^-1 ^in continuous fermentation at a dilution rate of 0.12 h^-1^, with 10 g l^-1 ^glucose and 10 g l^-1 ^xylose as carbon sources.

**Conclusion:**

The cofactor preference of *P. stipitis *XR was altered by site-directed mutagenesis. When the K270R XR was combined with a metabolic engineering strategy that ensures high xylose utilization capabilities, a recombinant *S. cerevisiae *strain was created that provides a unique combination of high xylose consumption rate, high ethanol yield and low xylitol yield during ethanolic xylose fermentation.

## Background

Xylose is the second most abundant carbohydrate in nature after glucose and one of the fermentable sugars in lignocellulosic biomass. *Saccharomyces cerevisiae *is the industrially most commonly used ethanol producer and *S. cerevisiae *strains have been genetically engineered to utilize xylose (recently reviewed in [1-3]).

In fungi, xylose catabolism begins with its conversion by xylose reductase (XR) and xylitol dehydrogenase (XDH) to xylulose, which after phosphorylation is assimilated via the non-oxidative pentose phosphate pathway. Anaerobic xylose fermentation by recombinant *S. cerevisiae *strains harbouring the XR-XDH pathway generally results in ethanol yields far below the theoretical 0.51 g g^-1 ^[4-8]. A significant fraction of the consumed xylose is secreted as xylitol, which has been ascribed to the difference in cofactor preference of the NAD(P)H-dependent XR and the NAD^+^-dependent XDH [4,9]. Xylitol formation can be limited by expressing a xylose isomerase (XI) instead of the XR-XDH pathway [10,11]. However, in a recent study, a strain carrying the *Pichia stipitis *XR-XDH pathway showed significantly higher xylose consumption rate and higher specific ethanol productivity compared with an isogenic strain carrying the *Pyromyces *XI pathway [12]. This indicates that an XR-XDH pathway engineered to be redox-neutral while maintaining the capability of high flux towards the central carbon metabolism could be the key to efficient anaerobic xylose fermentation.

High ethanol yield and low xylitol yield have been reported when mutated XR or XDH genes have been evaluated in xylose fermentation [13-20]. However, in these investigations, xylose utilization rates remained low which indicate limitations other than cofactor availability. Xylose utilization benefits from overexpression of genes encoding xylulokinase (XK) [6] and the four enzymes that constitute the non-oxidative pentose phosphate pathway (PPP), transaldolase, transketolase, ribose 5-phosphate ketol-isomerase and ribulose 5-phosphate epimerase [21-23]. In addition, deletion of the *GRE3 *gene, encoding an exclusively NADPH-dependent aldose reductase, decreases xylitol formation [24].

In the current study, genes encoding XRs with different cofactor affinities were expressed in *S. cerevisiae *strains with high xylose utilization capability due to overexpression of XK and the non-oxidative PPP. Minimized background NADPH-dependent aldose reductase activity was ensured by *GRE3 *gene deletion. The effect of different mutations in *P. stipitis *XR on the kinetic properties of the enzyme and on the xylose fermentation capability of the corresponding strains was evaluated. Additionally, the *C. parapsilosis XYL1 *gene [25], encoding an NADH-preferring XR, was expressed and evaluated in recombinant *S. cerevisiae*.

## Methods

### Strains, plasmids and medium

*Escherichia coli *strain DH5*α *(Life Technologies, Rockville, MD, USA) was used for cloning. Plasmids and *S. cerevisiae *strains are summarized in Table 1. All strains were stored in 15% glycerol at -80°C. *E. coli *was grown in LB-medium [26]. Yeast cells from freshly streaked yeast peptone dextrose (YPD) plates [26] or defined mineral medium plates [13] were used for inoculation. Liquid cultures of *S. cerevisiae *were grown in YPD medium [26] or defined mineral medium [13]. Defined mineral medium [13] supplemented with 0.4 g l^-1 ^Tween 80, 0.01 g l^-1 ^ergosterol and 0.5 ml l^-1 ^antifoam (Dow Corning^® ^Antifoam RD Emulsion, VWR International Ltd, Poole, UK) was used in anaerobic fermentation.

**Table 1:** Plasmids and *S. cerevisiae *strains used in this study.

**Plasmids and Strains**	**Relevant genotype**	**Reference**
pY7	*ADH1p-XYL1-ADH1t*, *PGK1p-XYL2-PGK1t*, *URA3*, 2 μ	[31]
YIplac211 PGK	*PGK1p-PGK1t, URA3*	[13]
YIplac211 PGK *XYL1*(K270M)	*PGK1p-XYL1*(K270M)-*PGK1t, URA3*	[13]
YIplac211 PGK *XYL1*(K270R)	*PGK1p-XYL1*(K270R)-*PGK1t, URA3*	This work
pUC57-CpXR	*XYL1*(*C. parapsilosis*)	This work
YIplac128	*LEU2*	[32]
YIplac211	*URA3*	[32]
YIpOB1	*ADH1p-XYL1-ADH1t*, *PGK1p-XYL2-PGK1t*, *LEU2*	This work
YIpOB2	*ADH1p-XYL1-ADH1t*, *PGK1p-XYL2-PGK1t*, *URA3*	This work
YIpOB3	*ADH1p-ADH1t*, *PGK1p-XYL2-PGK1t*, *URA3*	This work
YIpOB4	*ADH1p-XYL1*(K270M)-*ADH1t*, *PGK1p-XYL2-PGK1t*, *URA3*	This work
YIpOB5	*ADH1p-XYL1*(K270R)-*ADH1t*, *PGK1p-XYL2-PGK1t*, *URA3*	This work
YIpOB6	*ADH1p-XYL1*(*C. parapsilosis*)-*ADH1t*, *PGK1p-XYL2-PGK1t*, *URA3*	This work
TMB 3265	CEN.PK 113-11C, *MAT*a, *ura3-52*, *his3::HIS3 *YIpXDH/XK	[30]
TMB 3200	TMB 3265, *ura3::URA3 *YIplac211 PGK *XYL1*(K270R)	This work
TMB 3044	CEN.PK 2-1C, *MAT*a, *ura3*-*52*, *Δgre3*, *his3::HIS3 PGK1p-XKS1-PGK1t*, *TAL1::PGK1p-TAL1-PGK1t, TKL1::PGK1p-TKL1-PGK1t*, *RKI1::PGK1p-RKI1-PGK1t, RPE1::PGK1p-RPE1-PGK1t*	[22]
TMB 3321/Y-PsNative	TMB 3044, *ura3::URA3 *YIpOB2	This work
TMB 3322/Y-PsK270M	TMB 3044, *ura3::URA3 *YIpOB4	This work
TMB 3323/Y-PsK270R	TMB 3044, *ura3::URA3 *YIpOB5	This work
TMB 3324/Y-CpXR	TMB 3044, *ura3::URA3 *YIpOB6	This work

### Genetic techniques

Plasmid DNA was prepared with the GeneJET™ Plasmid Miniprep Kit (Fermentas UAB, Vilnius, Lithuania). Agarose gel DNA extraction was performed with QIAquick^® ^Gel Extraction Kit (Qiagen GmbH, Hilden, Germany). Primers from MWG-Biotech AG (Ebersberg, Germany) and *Pfu *DNA Polymerase and dNTP from Fermentas (Vilnius, Lithuania) were used for polymerase chain reactions (PCR). Primers used are listed in Table 2. PCR amplification was performed in a GeneAmp PCR system 9700 (Applied Biosystems, Foster City, CA, USA). PCR product purification was performed with the E.Z.N.A.^® ^Cycle-Pure Kit (Omega Bio-tek Inc, Doraville, GA, USA). BigDye^® ^Terminator v1.1 Cycle Sequencing Kit (Applied Biosystems) was used for DNA sequencing reactions. Sequencing was performed by BM labbet AB (Furulund, Sweden). Restriction endonucleases, Shrimp Alkaline Phosphatase and T4 DNA Ligase from Fermentas (Vilnius, Lithuania) were used for DNA manipulation. The *XYL1 *gene from *Candida parapsilosis *was commercially synthesized (GenScript Corp., Piscataway, NJ, USA) with codons optimized for *S. cerevisiae *expression.

**Table 2:** Primers used in this study.

**Primer**	**Sequence**	**Restriction Endonuclease**
5XYL1s	5'-GC**GGATCC***TCTAGA*ATGCCTT-3'	***Bam*HI**
3XYL1s	5'-TT**GGATCC***TCTAGA*TTAGACGAAG-3'	***Bam*HI**
5K270R	5'-CATCATTCCAAGGTCCAACACTG-3'	
3K270R	5'-CAGTGTTGGACCTTGGAATGATG-3'	
pY7-XR-for	5'-GC**AAGCTT***GGCGCGCC*GGGATCGAAGAAATGATGG-3'	***Hind*III**, *Asc*I
pY7-XR-rev	5'-CGCGCGCG**CTGCAG**GTGTGGAAGAACGATTACAAC-3'	***Pst*I**
pY7-XDH-for	5'-GC**CTGCAG**TCTAACTGATCTATCCAAAACTG-3'	***Pst*I**
pY7-XDH-rev	5'-CGT**GAGCTC***CGTACG*TAACGAACGCAGAATTTTC-3'	***Sac*I**, *Bsi*WI

Sites for restriction endonucleases are indicated in **bold **or *italic*. The codon giving the *P. stipitis *XR Arg at amino acid position 270 is underlined in primers 5K270R and 3K270R.

Competent *E. coli *DH5α cells were prepared and transformed as described elsewhere [27] and transformed *E. coli *strains were selected on LB plates [26] containing 100 mg l^-1 ^ampicillin (IBI Shelton Scientific, Inc., Shelton, CT). *E. coli *strains were grown in LB medium containing 100 mg l^-1 ^ampicillin for plasmid amplifications. Yeast strains were transformed with the lithium acetate method [28] and transformed yeast strains were selected for prototrophy on defined mineral medium plates containing 20 g l^-1 ^glucose.

### Construction of TMB 3200

The *P. stipitis XYL1 *gene carrying the K270R (Lys270Arg) mutation was generated by site-directed mutagenesis using the overlap extension PCR protocol [29]. In the first step, two separate PCR amplifications were done using plasmid YIplac211 PGK *XYL1*(K270M) [13] as template, primers 5XYL1s and 3K270R (Table 2) in one reaction mix and primers 5K270R and 3XYL1s (Table 2) in the other. Primers 3K270R and 5K270R are complementary to each other. In the second step, the two PCR products were mixed with primers 5XYL1s and 3XYL1s and fused together by PCR forming *XYL1*(K270R). The product was cut with *Bam*HI and inserted after the *PGK1 *promoter at the *Bgl*II site of YIplac211 PGK [13], resulting in YIplac211 PGK *XYL1*(K270R). The mutation was verified by sequencing. YIplac211 PGK *XYL1*(K270R) was cleaved with *Bpu*10I within the *URA3 *gene and transformed into TMB 3265 [30] resulting in TMB 3200.

### Construction of TMB 3321, TMB 3322, TMB 3323 and TMB 3324

Primers pY7-XR-for and pY7-XR-rev (Table 2) were used to amplify *ADH1p-XYL1-ADH1t *with PCR. Primers pY7-XDH-for and pY7-XDH-rev (Table 2) were used to amplify *PGK1p-XYL2-PGK1t*. Plasmid pY7 [31] was used as a template in both cases. *ADH1p-XYL1-ADH1t *was digested with *Hind*III and *Pst*I, and *PGK1p-XYL2-PGK1t *was digested with *Pst*I and *Sac*I. The resulting fragments were inserted into YIplac128 [32] creating YIpOB1. The DNA cassette containing *ADH1p-XYL1-ADH1t PGK1p-XYL2-PGK1t *was excised with *Hind*III and *Sac*I and inserted into YIplac211 [32] creating YIpOB2. The *XYL1 *gene was removed from YIpOB2 by digestion with *Xba*I and self-ligation to create YIpOB3. Plasmids YIplac211 PGK *XYL1*(K270M), YIplac211 PGK *XYL1*(K270R) and pUC57-CpXR were digested with *Xba*I and the *XYL1*(K270M), *XYL1*(K270R) and *XYL1*(*C. parapsilosis*) fragments were inserted into the *Xba*I site of YIpOB3, resulting in YIpOB4, YIpOB5 and YIpOB6, respectively. Correct orientation and sequence of the inserts were verified by restriction analysis and sequencing. YIpOB2, YIpOB4, YIpOB5 and YIpOB6 were cleaved with *Apa*I within the *URA3 *gene and transformed into TMB 3044 [22]. This resulted in strains TMB 3321, TMB 3322, TMB 3323 and TMB 3324, respectively, henceforth referred to as Y-PsNative, Y-PsK270M, Y-PsK270R and Y-CpXR.

### Batch fermentation

Anaerobic batch fermentation was carried out in 3-litre ADI Autoclavable Bio Reactor Systems (Applikon, Schiedam, The Netherlands) with a working volume of 1 litre. Cells were pre-cultivated in shake flasks in defined mineral medium with 20 g l^-1 ^glucose, washed with sterile water and inoculated into the bioreactor to an optical density at 620 nm (OD620) of 0.2. Defined mineral medium with doubled concentration of all salts, trace elements and vitamins, containing 20 g l^-1 ^glucose and 50 g l^-1 ^xylose, was used. The temperature was 30°C, stirring was set to 200 rpm and pH 5.5 was maintained with 3 M KOH. Anaerobic conditions were attained by sparging with nitrogen gas containing less than 5 ppm O_2 _(AGA GAS AB, Sundbyberg, Sweden) before inoculation. During fermentation, anaerobic conditions were maintained by the produced CO_2 _that diffused through a water lock. The experiments were performed at least in biological duplicates.

### Continuous fermentation

Continuous fermentation was conducted anaerobically in 2-litre Biostat^® ^A bioreactors (B. Braun Biotech International, Melsungen, Germany) with a working volume of 1 litre. Defined mineral medium with 10 g l^-1 ^glucose and 10 g l^-1 ^xylose was used for pre-cultivation and continuous fermentation. Cells pre-cultivated in shake flasks and washed with sterile water were used to inoculate the bioreactor to an OD620 of 0.2. Continuous fermentation at dilution rates of 0.06 and 0.12 h^-1 ^was started after glucose depletion. The temperature was 30°C, stirring 200 rpm and pH 5.5 was maintained with 3 M KOH. Anaerobic conditions were obtained by sparging with nitrogen gas containing less than 5 ppm O_2 _(AGA GAS AB, Sundbyberg, Sweden) at a constant gas flow of 0.2 litre min^-1 ^controlled by mass flow meters (Bronkhorst HI-TEC, Ruurlo, the Netherlands). The off-gas condensers were cooled to 4°C, and the medium reservoirs were continuously sparged with nitrogen gas. Steady-state was assumed after five residence times and verified by measurements of cell density and CO_2 _production. The experiments were performed in biological duplicates.

### Analyses

Growth was determined by measuring OD620 with a Hitachi U-1800 Spectrophotometer (Hitachi Ltd., Tokyo, Japan). Concentration of glucose, xylose, xylitol, glycerol, pyruvate, acetate, ethanol and succinate was determined by high-performance liquid chromatography (Waters, Milford, MA, USA) with an Aminex HPX-87 H ion exchange column (Bio-Rad, Hercules, CA, USA), refractive index detector (RID-6A, Shimadzu, Kyoto, Japan) and UV detector (2487, Waters). The mobile phase was 5 mM H_2_SO_4_, temperature 45°C and flow rate 0.6 ml min^-1^. The composition of the outgoing gas was monitored by a Carbon Dioxide and Oxygen Monitor Type 1308 (Brüel & Kjær, Copenhagen, Denmark). Cell dry weight was determined in triplicate by filtering a known volume of culture broth through 0.45 μm Supor^® ^450 Membrane filters (Pall Life Sciences, Ann Arbor, MI, USA), after which the filters were dried in a microwave oven and weighed. The fractions of protein, polysaccharides [33] and RNA [34] in the biomass were determined at steady-state in continuous fermentation. A previously developed stoichiometric model [35] was used to estimate the intracellular carbon fluxes at steady-state in continuous fermentation.

### Ethanol Evaporation

Ethanol evaporation was determined experimentally for the setup used for continuous fermentation. Ethanol was added to a fermentor sparged with a nitrogen gas flow of 0.2 litre min^-1 ^and the ethanol concentration was measured over time. The evaporation rate followed Equation (1) with a proportionality constant of *k *= 0.004.

(1)

Ethanol evaporation was estimated for each continuous fermentation and constitutes together with ethanol measured by HPLC the total ethanol production.

### Enzymatic activity

Strains were cultivated for enzyme activity measurements in defined mineral medium containing 20 g l^-1 ^glucose and harvested in the exponential growth phase. Cells were washed with sterile water and treated with yeast protein extraction solution Y-PER (Pierce, Rockford, IL, USA). Coomassie Protein Assay Reagent (Pierce) was used to determine protein concentration with Albumin Standard (Pierce). NAD(P)H-dependent XR activity was determined using an Ultrospec 2100 pro spectrophotometer (Amersham Biosciences, Uppsala, Sweden) operating at 30°C and 340 nm (*ε*_NAD(P)H _= 6220 M^-1 ^cm^-1^). Triethanolamine buffer (100 mM, pH 7.0) was used and reactions were started by addition of xylose. Crude extracts from strains Y-PsNative, Y-PsK270M, Y-PsK270R and Y-CpXR were assayed for functional XR expression using a standard assay with 200 μM NAD(P)H and 350 mM xylose as previously described [7]. XR kinetics in crude extracts from strains Y-PsNative, Y-PsK270M and Y-PsK270R were determined, with concentrations of xylose and NAD(P)H varied from less than half to more than five times the respective apparent *K*_m _value. The initial rates were fitted by unconstrained non-linear optimization in MatLab R2006a to Equation (2), which describes the initial rate for a two-substrate reaction following a compulsory-order ternary-complex mechanism [36].

(2)

*V*_max _is the maximum velocity, [A] and [B] are the concentrations of NAD(P)H and xylose, respectively, *K*_mA _and *K*_mB _are the Michaelis constants of NAD(P)H and xylose, respectively, and *K*_iA _is the dissociation constant of NAD(P)H.

## Results

### Strain construction

XR encoded by the *C. parapsilosis XYL1 *gene is the first XR enzyme reported to prefer NADH [25]. The *C. parapsilosis *XR carries an arginine instead of a lysine in the Ile-Pro-Lys-Ser motif that is conserved among NADPH-dependent xylose reductases [37]. The replacement of the lysine by an arginine in the K270R mutant of *P. stipitis *XR was made to mimic the *C. parapsilosis *XR. Strain TMB 3200 expressing the K270R mutant of *P. stipitis *XR (Table 1) was constructed to assess the influence of the mutation on xylose fermentation by recombinant *S. cerevisiae*. The strain was compared in anaerobic continuous fermentation with TMB 3001 [7], which carries the native *P. stipitis *XR, XDH and overexpressed endogenous XK. Increased ethanol yield and decreased xylitol yield was observed but the xylose utilization rate was not improved (results not shown). It was suspected that the xylose utilization rate was limited by other factors than the cofactor imbalance caused by the NAD(P)H-dependent XR and the strictly NAD^+^-dependent XDH.

Overexpression of XK together with the non-oxidative PPP improved xylose utilization by recombinant *S. cerevisiae *[22,23]. Also, the deletion of the endogenous aldose reductase *GRE3 *minimized background XR activity and decreased xylitol formation [24]. Four isogenic CEN.PK-based strains (Table 1) with these features were constructed to evaluate how the kinetic properties of XR affect xylose fermentation by recombinant *S. cerevisiae*. Strain Y-PsNative carrying the native *P. stipitis *XR served as a reference strain. Y-PsK270M contained the K270M mutant of *P. stipitis *XR that previously has been shown to reduce xylitol yield and increase ethanol yield in xylose fermentation [13]. Y-PsK270R expressed the K270R mutant of *P. stipitis *XR and Y-CpXR contained a synthetic *C. parapsilosis XYL1 *gene [25] that had been codon-optimized for *S. cerevisiae *expression.

### Enzyme activities and kinetic properties

Crude extracts of strains Y-PsNative, Y-PsK270M, Y-PsK270R and Y-CpXR were analyzed for functional XR expression with a standard assay (200 μM NAD(P)H, 350 mM xylose) (Table 3). Y-PsK270M displayed only about 34% and 36% of the NADPH and NADH-dependent XR activities compared with the reference strain Y-PsNative. In contrast, Y-PsK270R showed 2.4-fold and 3.2-fold higher NADPH and NADH-dependent XR activities compared with Y-PsNative. Y-CpXR with the *C. parapsilosis XYL1 *did not display any significant NADPH or NADH-dependent XR activity. The two *P. stipitis *XR mutants displayed no change of cofactor preference compared with the native XR under standard assay conditions (Table 3).

**Table 3:** Enzyme activities and kinetic properties

**Strain**	**XR gene**	**Cofactor**	**Specific XR activity U mg^-1 ^protein**	***K*_mA _μM**	***K*_mB _mM**	***K*_iA _μM**	***V*_max _U mg^-1 ^protein**
Y-PsNative	*XYL1*	NADPH NADH	0.23 ± 0.06 0.10 ± 0.02	1.0 ± 0.6 28.7 ± 5.4	62.2 ± 27.7 59.2 ± 10.5	1.4 ± 1.2 25.9 ± 11.7	0.30 ± 0.05 0.21 ± 0.01
Y-PsK270M	*XYL1*(K270M)	NADPH NADH	0.08 ± 0.01 0.04 ± 0.01	290 ± 78.6 -	454 ± 142 -	293 ± 169 -	0.91 ± 0.09 -
Y-PsK270R	*XYL1*(K270R)	NADPH NADH	0.54 ± 0.02 0.32 ± 0.02	25.8 ± 9.1 62.8 ± 18.7	468 ± 151 145 ± 36.9	22.9 ± 17.6 57.4 ± 34.9	2.13 ± 0.24 0.96 ± 0.09
Y-CpXR	*XYL1*(*C. parapsilosis*)	NADPH NADH	n.d. n.d.	- -	- -	- -	- -

Specific XR activity in cell extracts from strains Y-PsNative, Y-PsK270M, Y-PsK270R and Y-CpXR in standard conditions (200 μM NAD(P)H, 350 mM xylose) and estimated kinetic parameters for NAD(P)H reduction of xylose by corresponding cell extracts. *K*_mA _and *K*_mB _are the Michaelis constants of NAD(P)H and xylose, respectively, *K*_iA _is the dissociation constant of NAD(P)H and *V*_max _is the maximum velocity.

n.d. not detected

- not determined

A kinetic study was made on crude extracts from strains Y-PsNative, Y-PsK270M and Y-PsK270R. The data was fitted to Equation (2) and the resulting constants are summarized in Table 3. Compared with native XR from *P. stipitis*, the K270M mutation resulted in a significant increase in the *K*_m _values for both NADPH and NADH. In fact, the kinetic parameters for the NADH-linked reaction catalyzed by the K270M mutant could not even be determined since this mutant could not be saturated with NADH. The K270R mutation increased the *K*_m _value for NADPH 25-fold, while the *K*_m _for NADH only increased two-fold.

### Batch fermentation

**Figure 1:**
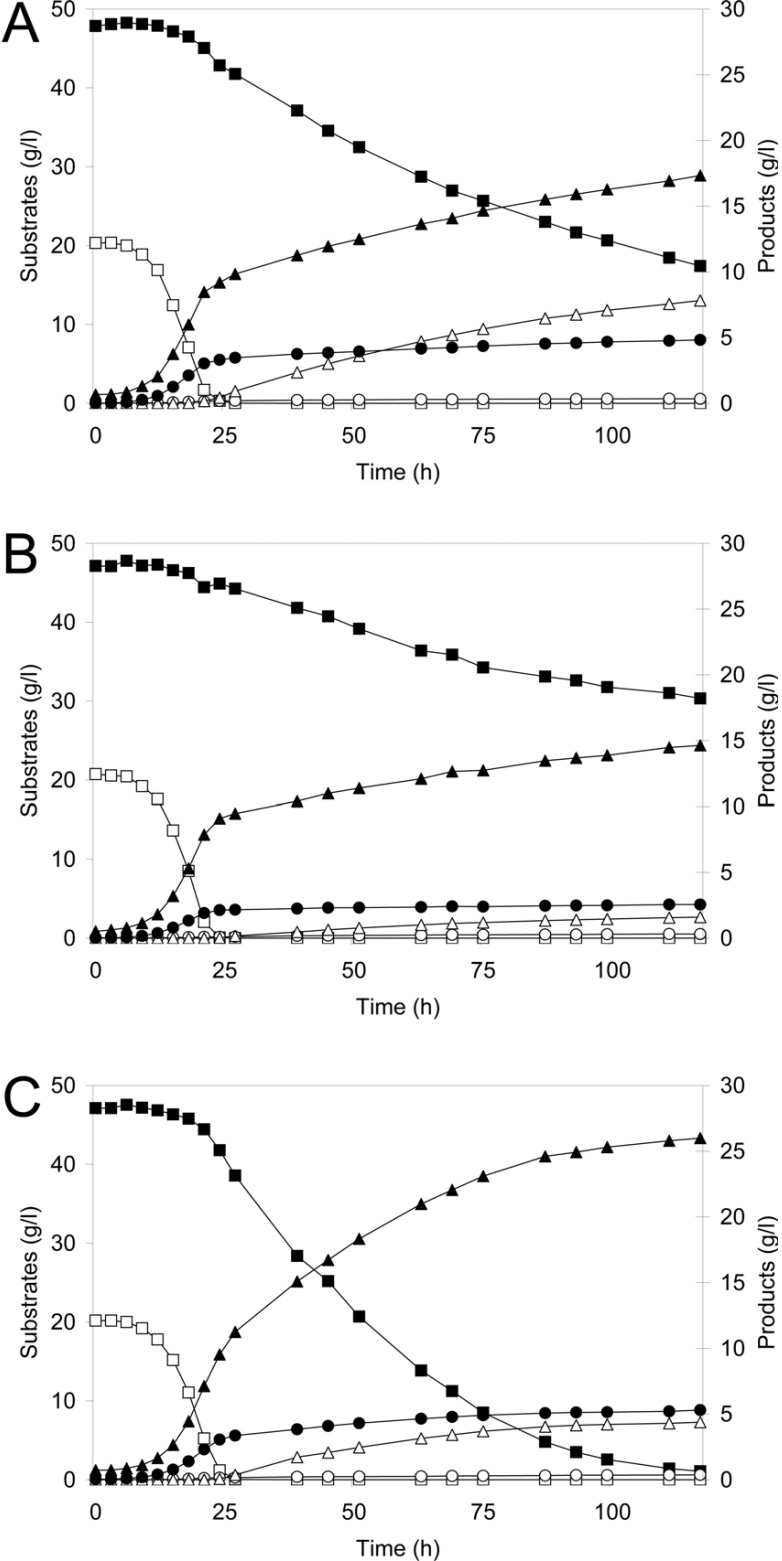
**Anaerobic batch fermentation of 20 g l^-1 ^glucose and 50 g l^-1 ^xylose**. Strains: Y-PsNative (A); Y-PsK270M (B); and Y-PsK270R (C). Symbols: black square – xylose, open square – glucose, black triangle – ethanol, open triangle – xylitol, black circle – glycerol, open circle – acetate.

Strains Y-PsNative, Y-PsK270M and Y-PsK270R were compared in anaerobic batch fermentation with 20 g l^-1 ^glucose and 50 g l^-1 ^xylose (Figure 1). Table 4 summarizes xylose consumption, ethanol concentration and product yields after 117 h of fermentation. The reference strain Y-PsNative consumed 30.4 g l^-1 ^xylose and produced 16.7 g l^-1 ^ethanol while Y-PsK270R consumed 46.1 g l^-1 ^xylose and produced 25.3 g l^-1 ^ethanol. Y-PsK270M consumed the least xylose (16.8 g l^-1^) and produced the lowest ethanol concentration (14.1 g l^-1^) of the three strains. The reference strain Y-PsNative produced an ethanol yield of 0.33 g ethanol (g consumed sugars)^-1 ^and a xylitol yield of 0.26 g xylitol (g consumed xylose)^-1^. Both strains with mutated XR produced higher ethanol yields (0.38 g ethanol (g consumed sugars)^-1^) and significantly lower xylitol yields (0.09 g xylitol (g consumed xylose)^-1^) than the reference strain.

**Table 4:** Batch fermentation

**Strain**	**Xylose** **consumed** **(g l^-1^)**	**Ethanol** **produced** **(g l^-1^)**	**Yields (g product (g consumed sugars)^-1^)**				
			
			**Ethanol**	**Xylitol^a^**	**Glycerol**	**Biomass**	**Acetate**
Y-PsNative	30.4 ± 2.3	16.7 ± 0.4	0.33 ± 0.02	0.26 ± 0.03	0.095 ± 0.001	0.040 ± 0.001	0.011 ± 0.002
Y-PsK270M	16.8 ± 0.2	14.1 ± 0.3	0.38 ± 0.01	0.09 ± 0.01	0.067 ± 0.000	0.054 ± 0.001	0.013 ± 0.001
Y-PsK270R	46.1 ± 1.3	25.3 ± 0.5	0.38 ± 0.01	0.09 ± 0.01	0.079 ± 0.001	0.050 ± 0.001	0.009 ± 0.000

Xylose consumption, ethanol production and product yields after 117 hours (see Figure 1) anaerobic batch fermentation of 20 g l^-1 ^glucose and 50 g l^-1 ^xylose by strains Y-PsNative, Y-PsK270M and Y-PsK270R.

^a ^(g xylitol (g consumed xylose)^-1^)

### Continuous fermentation and flux analysis

Y-PsNative and Y-PsK270R were compared in anaerobic continuous fermentation with a feed containing 10 g l^-1 ^glucose and 10 g l^-1 ^xylose (Table 5). The continuous fermentation results were generally in agreement with the batch fermentation results (Table 4). Y-PsK270R gave 4% higher ethanol yields than Y-PsNative at both dilution rates. Y-PsK270R showed 17% and 9% higher specific xylose consumption rates and gave 60% and 78% lower xylitol yields compared with the reference strain Y-PsNative at dilution rates 0.06 h^-1 ^and 0.12 h^-1 ^respectively. Y-PsK270R also gave 17% and 22% lower glycerol yields than Y-PsNative at dilution rates 0.06 h^-1 ^and 0.12 h^-1 ^respectively.

**Table 5:** Continuous fermentation

**Dilution rate** **(h^-1^)**	**Strain**	**Specific consumption and production rates (g (g biomass)^-1 ^h^-1^)**			**Yields (g product (g consumed sugars)^-1^)**				**Carbon balance (%)**
				
		**Glucose**	**Xylose**	**Ethanol**	**Ethanol**	**Xylitol^a^**	**Glycerol**	**Biomass**	
0.06	Y-PsNative	-0.64 ± 0.02	-0.19 ± 0.01	0.31 ± 0.00	0.37 ± 0.02	0.30 ± 0.02	0.09 ± 0.00	0.07 ± 0.00	96 ± 2
	Y-PsK270R	-0.52 ± 0.01	-0.22 ± 0.01	0.29 ± 0.01	0.39 ± 0.00	0.12 ± 0.01	0.07 ± 0.01	0.08 ± 0.00	94 ± 1
									
0.12	Y-PsNative	-1.09 ± 0.03	-0.26 ± 0.02	0.50 ± 0.01	0.37 ± 0.02	0.24 ± 0.04	0.09 ± 0.01	0.09 ± 0.00	95 ± 3
	Y-PsK270R	-1.04 ± 0.06	-0.28 ± 0.01	0.51 ± 0.03	0.39 ± 0.01	0.05 ± 0.02	0.07 ± 0.01	0.09 ± 0.00	93 ± 1

Specific consumption (negative) and production (positive) rates, product yields and carbon balances in continuous fermentation of strains Y-PsNative and Y-PsK270R under anaerobic conditions at dilution rates of 0.06 h^-1 ^and 0.12 h^-1 ^with 10 g l^-1 ^glucose and 10 g l^-1 ^xylose.

^a ^(g xylitol (g consumed xylose)^-1^)

**Figure 2:**
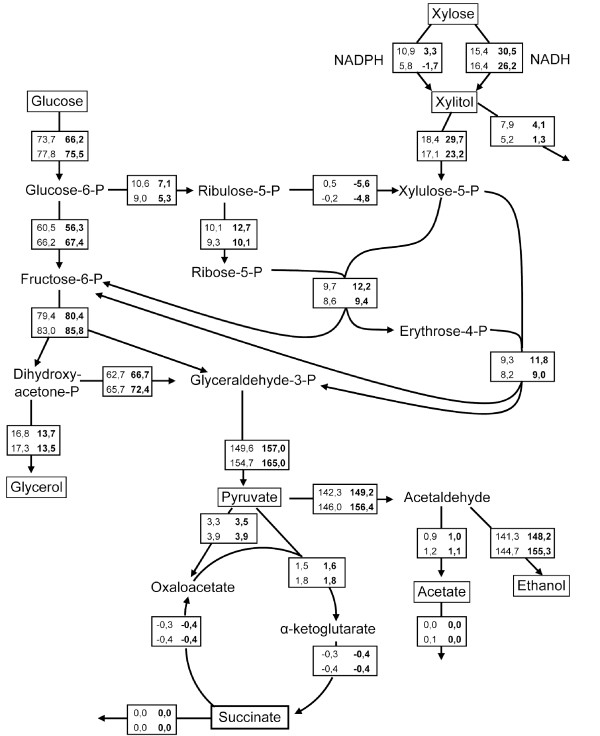
**Metabolic flux analysis**. Estimated metabolic fluxes in Y-PsNative and Y-PsK270R (bold) from continuous fermentation at dilution rate 0.06 h^-1 ^(upper values) and 0.12 h^-1 ^(lower values) with a feed containing 10 g l^-1 ^glucose and 10 g l^-1 ^xylose. All fluxes are normalized to a total specific sugar consumption of 100 mmol g^-1 ^biomass h^-1^. Substances shown inside boxes are substrates or products measured with high performance liquid chromatography.

The metabolic fluxes through Y-PsNative and Y-PsK270R were estimated (Figure 2) using a stoichiometric model [35]. The flux values were normalized to a total specific sugar consumption of 100 mmol g^-1 ^biomass h^-1^. The xylose fraction of the total specific sugar consumption was smaller for both strains at dilution rate 0.12 h^-1 ^compared with 0.06 h^-1^. According to the model, Y-PsK270R utilized a larger fraction of NADH in the XR reaction (90% and 100%) than Y-PsNative (59% and 74%) at dilution rates 0.06 h^-1 ^and 0.12 h^-1 ^respectively. The model also predicts that a smaller fraction of glucose-6-phospate enters the oxidative PPP in Y-PsK270R (11% and 7%) than in Y-PsNative (14% and 12%) at dilution rates 0.06 h^-1 ^and 0.12 h^-1 ^respectively.

## Discussion

This is the first time that an efficient xylose fermenting *S. cerevisiae *strain has been generated by a targeted metabolic engineering strategy, and where the XR-XDH xylose utilization pathway was chromosomally integrated to ensure stable expression of all required components (Table 6) [38]. Expression of K270R XR proved to be superior to expression of native XR in both batch and continuous fermentation. When compared with other strains in anaerobic continuous fermentation with 10 g l^-1 ^glucose and 10 g l^-1 ^xylose (Table 6), strain Y-PsK270R uniquely combines high xylose consumption rate and high ethanol yield due to low xylitol production. Strains TMB3270 and TMB3271, harbouring XR with the K270M mutation, gave lower xylitol yields compared with their reference strains TMB3001 and TMB3260 (Table 6) [13]. However, the K270M mutation also decreased the xylose consumption rate. Improved xylose utilization capability has previously been reported for strains TMB3400 and C1, generated by random mutagenesis and evolutionary engineering, respectively (Table 6) [39,40]. However, the exact mutations that caused the improvements are not known, and it is therefore difficult to transfer these traits. In addition, strains TMB3400 and C1 produce relatively high xylitol yields (Table 6). Effective ethanolic xylose fermentation in batch has been reported for XI strains, harbouring *Piromyces *XI on a multicopy plasmid [23,41]. When the *Piromyces *XI gene was chromosomally integrated in the strain background used for constructing Y-PsK270R, aerobic xylose growth could not be achieved [12]. In contrast, strain Y-PsK270R harbours all components required for effective xylose utilization chromosomally integrated. This ensures stable expression, and facilitates the transfer of these traits into an industrial strain.

**Table 6:** Strain comparison

**Strain**	**Relevant genotype**	**Dilution rate**	**Xylose consumption rate**	**Ethanol yield**	**Xylitol yield**	**Reference**
TMB3001	*XYL1*, *XYL2*, *XKS1*	0.06	0.12	0.37	0.52	[13]
TMB3260	(2 × *XYL1*), *XYL2*, *XKS1*	0.06	0.19	0.36	0.58	[13]
TMB3270	*XYL1*(K270M), *XYL2*, *XKS1*	0.06	0.05	0.40	0.31	[13]
TMB3271	(2 × *XYL1*(K270M)), *XYL2*, *XKS1*	0.06	0.16	0.40	0.44	[13]
TMB3400	*XYL1*, *XYL2*, *XKS1*, Mutated TMB3399	0.06	0.22	0.35	0.19	[1]
C1	*XYL1*, *XYL2*, *XKS1*, Evolved TMB3001	0.05	0.31	0.27^a^	0.35^a^	[38]
Y-PsNative	*XYL1*, *XYL2*, *XKS1*, *ΔGRE3*, overexpressed non-oxidative PPP	0.06	0.19	0.37	0.30	This work
Y-PsK270R	*XYL1*(K270R), *XYL2*, *XKS1*, *ΔGRE3*, overexpressed non-oxidative PPP	0.06	0.22	0.39	0.12	This work

Xylose consumption rate (g (g biomass)^-1 ^h^-1^), ethanol yield (g (g consumed sugars)^-1^) and xylitol yield (g (g consumed xylose)^-1^) in anaerobic continuous fermentation with recombinant *S. cerevisiae *strains, 10 g l^-1 ^glucose and 10 g l^-1 ^xylose.

^a ^Recalculated

In another fermentation study, the native *Candida tenuis *XR has been compared with its K274R-N276D double mutant in recombinant *S. cerevisiae *strains also expressing XDH from *Galactocandida mastotermitis *and overexpressing the endogenous XK gene [19]. In contrast to the current results, the double mutant *C. tenuis *XR did not improve the xylose uptake rate, suggesting that the high xylose utilization background of the strains used in the present study is required to make a full examination of a xylose utilization pathway.

The metabolic flux analysis indicated that the K270R XR utilizes a larger fraction of NADH compared with the native XR *in vivo*. This, in turn, made more NAD^+ ^available for the XDH reaction, and resulted in higher xylose consumption rate and lower xylitol production. Both strains carrying mutated XRs also gave lower glycerol yields in anaerobic batch fermentation compared with the reference strain. This is a further indication that NAD^+ ^is more available for the XDH reaction in these strains since glycerol formation is the main NAD^+ ^generation pathway in *S. cerevisiae *under anaerobic conditions [42-44].

The estimated kinetic parameters of the native *P. stipitis *XR correspond well to previously published data [45]. The K270M and K270R mutations affect the kinetic properties of the enzyme similarly to the corresponding mutations in *C. tenuis *XR [46]. According to the metabolic flux analysis, the K270R XR appears to use more NADH than NADPH *in vivo*, even though the *K*_m _value for NADPH is estimated to be around half of the *K*_m _value for NADH. This suggests that that the intracellular level of NADH is much higher than the intracellular level of NADPH. The K270M mutation affected both NADPH and NADH affinity in xylose reduction, in contrast to the glyceraldehyde reduction where the apparent affinity for NADH remained unchanged [47]. The K270M mutation reduced the xylose utilization rate, which agrees with previous observations [13]. Crude extract from Y-PsK270M displayed lower XR activity in standard assay conditions which indicated lower expression of the K270M XR. However, the kinetic study revealed that the K270M XR has a lower affinity for NAD(P)H and xylose. The K270M XR was far from saturated in the standard assay conditions, which explains the lower XR activities.

## Conclusion

The cofactor preference of *P. stipitis *XR was altered by site-directed mutagenesis. When the K270R XR was combined with a metabolic engineering strategy that ensures high xylose utilization capabilities, a recombinant *S. cerevisiae *strain was created that provides a unique combination of high xylose consumption rate, high ethanol yield and low xylitol yield during ethanolic xylose fermentation.

## Competing interests

The authors declare that they have no competing interests.

## Authors' contributions

OB participated in the design of the study, performed the experimental work and wrote the manuscript. BHH participated in the design of the study and commented on the manuscript. MFGG participated in the design of the study and commented on the manuscript. All the authors read and approved the final manuscript.

## References

[B1] Hahn-Hägerdal B, Karhumaa K, Jeppsson M, Gorwa-Grauslund MF (2007). Metabolic engineering for pentose utilization in *Saccharomyces cerevisiae*. Adv Biochem Eng Biotechnol.

[B2] van Maris AJ, Winkler AA, Kuyper M, de Laat WT, van Dijken JP, Pronk JT (2007). Development of efficient xylose fermentation in *Saccharomyces cerevisiae*: xylose isomerase as a key component. Adv Biochem Eng Biotechnol.

[B3] Chu BC, Lee H (2007). Genetic improvement of *Saccharomyces cerevisiae *for xylose fermentation. Biotechnol Adv.

[B4] Kötter P, Ciriacy M (1993). Xylose fermentation by *Saccharomyces cerevisiae*. Appl Microbiol Biotechnol.

[B5] Tantirungkij M, Nakashima N, Seki T, Yoshida T (1993). Construction of xylose-assimilating *Saccharomyces cerevisiae*. J Ferm Bioeng.

[B6] Ho NW, Chen Z, Brainard AP (1998). Genetically engineered *Saccharomyces *yeast capable of effective cofermentation of glucose and xylose. Appl Environ Microbiol.

[B7] Eliasson A, Christensson C, Wahlbom CF, Hahn-Hägerdal B (2000). Anaerobic xylose fermentation by recombinant *Saccharomyces cerevisiae *carrying *XYL1*, *XYL2*, and *XKS1 *in mineral medium chemostat cultures. Appl Environ Microbiol.

[B8] Toivari MH, Aristidou A, Ruohonen L, Penttilä M (2001). Conversion of xylose to ethanol by recombinant *Saccharomyces cerevisiae*: Importance of xylulokinase (*XKS1*) and oxygen availability. Metab Eng.

[B9] Bruinenberg PM, de Bot PHM, van Dijken JP, Scheffers WA (1984). NADH-linked aldose reductase: the key to anaerobic alcoholic fermentation of xylose by yeasts. Appl Microbiol Biotechnol.

[B10] Walfridsson M, Bao X, Anderlund M, Lilius G, Bülow L, Hahn-Hägerdal B (1996). Ethanolic fermentation of xylose with *Saccharomyces cerevisiae *harboring the *Thermus thermophilus xylA *gene, which expresses an active xylose (glucose) isomerase. Appl Environ Microbiol.

[B11] Kuyper M, Harhangi HR, Stave AK, Winkler AA, Jetten MS, de Laat WT, den Ridder JJ, Op den Camp HJ, van Dijken JP, Pronk JT (2003). High-level functional expression of a fungal xylose isomerase: the key to efficient ethanolic fermentation of xylose by *Saccharomyces cerevisiae*?. FEMS Yeast Res.

[B12] Karhumaa K, Sanchez RG, Hahn-Hägerdal B, Gorwa-Grauslund MF (2007). Comparison of the xylose reductase-xylitol dehydrogenase and the xylose isomerase pathways for xylose fermentation by recombinant *Saccharomyces cerevisiae*. Microb Cell Fact.

[B13] Jeppsson M, Bengtsson O, Franke K, Lee H, Hahn-Hägerdal B, Gorwa-Grauslund MF (2006). The expression of a *Pichia stipitis *xylose reductase mutant with higher K(M) for NADPH increases ethanol production from xylose in recombinant *Saccharomyces cerevisiae*. Biotechnol Bioeng.

[B14] Saleh AA, Watanabe S, Annaluru N, Kodaki T, Makino K (2006). Construction of various mutants of xylose metabolizing enzymes for efficient conversion of biomass to ethanol. Nucleic Acids Symp Ser (Oxf).

[B15] Watanabe S, Pack SP, Saleh AA, Annaluru N, Kodaki T, Makino K (2007). The positive effect of the decreased NADPH-preferring activity of xylose reductase from *Pichia stipitis *on ethanol production using xylose-fermenting recombinant *Saccharomyces cerevisiae*. Biosci Biotechnol Biochem.

[B16] Watanabe S, Saleh AA, Pack SP, Annaluru N, Kodaki T, Makino K (2007). Ethanol production from xylose by recombinant *Saccharomyces cerevisiae *expressing protein engineered NADP+-dependent xylitol dehydrogenase. J Biotechnol.

[B17] Watanabe S, Abu Saleh A, Pack SP, Annaluru N, Kodaki T, Makino K (2007). Ethanol production from xylose by recombinant *Saccharomyces cerevisiae *expressing protein-engineered NADH-preferring xylose reductase from *Pichia stipitis*. Microbiology.

[B18] Matsushika A, Watanabe S, Kodaki T, Makino K, Sawayama S (2008). Bioethanol production from xylose by recombinant *Saccharomyces cerevisiae *expressing xylose reductase, NADP(+)-dependent xylitol dehydrogenase, and xylulokinase. J Biosci Bioeng.

[B19] Petschacher B, Nidetzky B (2008). Altering the coenzyme preference of xylose reductase to favor utilization of NADH enhances ethanol yield from xylose in a metabolically engineered strain of *Saccharomyces cerevisiae*. Microb Cell Fact.

[B20] Dmytruk OV, Dmytruk KV, Abbas CA, Voronovsky AY, Sibirny AA (2008). Engineering of xylose reductase and overexpression of xylitol dehydrogenase and xylulokinase improves xylose alcoholic fermentation in the thermotolerant yeast *Hansenula polymorpha*. Microb Cell Fact.

[B21] Johansson B, Hahn-Hägerdal B (2002). The non-oxidative pentose phosphate pathway controls the fermentation rate of xylulose but not of xylose in *Saccharomyces cerevisiae *TMB3001. FEMS Yeast Res.

[B22] Karhumaa K, Hahn-Hägerdal B, Gorwa-Grauslund MF (2005). Investigation of limiting metabolic steps in the utilization of xylose by recombinant *Saccharomyces cerevisiae *using metabolic engineering. Yeast.

[B23] Kuyper M, Hartog MM, Toirkens MJ, Almering MJ, Winkler AA, van Dijken JP, Pronk JT (2005). Metabolic engineering of a xylose-isomerase-expressing *Saccharomyces cerevisiae *strain for rapid anaerobic xylose fermentation. FEMS Yeast Res.

[B24] Träff KL, Otero Cordero RR, van Zyl WH, Hahn-Hägerdal B (2001). Deletion of the *GRE3 *Aldose Reductase Gene and Its Influence on Xylose Metabolism in Recombinant Strains of *Saccharomyces cerevisiae *Expressing the *xylA *and *XKS1 *Genes. Appl Environ Microbiol.

[B25] Lee JK, Koo BS, Kim SY (2003). Cloning and characterization of the *xyl1 *gene, encoding an NADH-preferring xylose reductase from *Candida parapsilosis*, and its functional expression in *Candida tropicalis*. Appl Environ Microbiol.

[B26] Ausubel FM, Brent R, Kingston E, Moore DD, Seidman JG, Smith JA, Struhl K (1995). Current protocols in molecular biology.

[B27] Inoue H, Nojima H, Okayama H (1990). High efficiency transformation of *Escherichia coli *with plasmids. Gene.

[B28] Güldener U, Heck S, Fielder T, Beinhauer J, Hegemann JH (1996). A new efficient gene disruption cassette for repeated use in budding yeast. Nucleic Acids Res.

[B29] Ho SN, Hunt HD, Horton RM, Pullen JK, Pease LR (1989). Site-directed mutagenesis by overlap extension using the polymerase chain reaction. Gene.

[B30] Träff-Bjerre KL, Jeppsson M, Hahn-Hägerdal B, Gorwa-Grauslund MF (2004). Endogenous NADPH-dependent aldose reductase activity influences product formation during xylose consumption in recombinant *Saccharomyces cerevisiae*. Yeast.

[B31] Walfridsson M, Anderlund M, Bao X, Hahn-Hägerdal B (1997). Expression of different levels of enzymes from the *Pichia stipitis XYL1 *and *XYL2 *genes in *Saccharomyces cerevisiae *and its effects on product formation during xylose utilisation. Appl Microbiol Biotechnol.

[B32] Gietz RD, Sugino A (1988). New yeast-*Escherichia coli *shuttle vectors constructed with in vitro mutagenized yeast genes lacking six-base pair restriction sites. Gene.

[B33] Herbert D, Phipps P, Strange R (1971). Chemical analysis of microbial cells. Methods Microbiol.

[B34] Benthin S, Nielsen J, Villadsen J (1991). A simple and reliable method for the determination of cellular RNA content. Biotechnol Tech.

[B35] Wahlbom CF, Eliasson A, Hahn-Hägerdal B (2001). Intracellular fluxes in a recombinant xylose-utilizing *Saccharomyces cerevisiae *cultivated anaerobically at different dilution rates and feed concentrations. Biotechnol Bioeng.

[B36] Cornish-Bowden A (2004). Fundamentals of enzyme kinetics.

[B37] Lee H (1998). The structure and function of yeast xylose (aldose) reductases. Yeast.

[B38] Sonderegger M, Jeppsson M, Hahn-Hägerdal B, Sauer U (2004). Molecular basis for anaerobic growth of *Saccharomyces cerevisiae *on xylose, investigated by global gene expression and metabolic flux analysis. Appl Environ Microbiol.

[B39] Wahlbom CF, van Zyl WH, Jönsson LJ, Hahn-Hägerdal B, Otero RR (2003). Generation of the improved recombinant xylose-utilizing *Saccharomyces cerevisiae *TMB 3400 by random mutagenesis and physiological comparison with *Pichia stipitis *CBS 6054. FEMS Yeast Res.

[B40] Sonderegger M, Sauer U (2003). Evolutionary Engineering of *Saccharomyces cerevisiae *for Anaerobic Growth on Xylose. Appl Environ Microbiol.

[B41] Kuyper M, Winkler AA, van Dijken JP, Pronk JT (2004). Minimal metabolic engineering of *Saccharomyces cerevisiae *for efficient anaerobic xylose fermentation: a proof of principle. FEMS Yeast Res.

[B42] Oura E (1977). Reaction products of yeast fermentation. Process Biochem.

[B43] van Dijken JP, Scheffers WA (1986). Redox balances in the metabolism of sugars by yeast. FEMS Microbiol Rev.

[B44] Albers E, Larsson C, Lidén G, Niklasson C, Gustafsson L (1996). Influence of the nitrogen source on *Saccharomyces cerevisiae *anaerobic growth and product formation. Appl Environ Microbiol.

[B45] Rizzi M, Erlemann P, Bui-Thanh NA, Dellweg H (1988). Xylose fermentation by yeast. 4. Purification and kinetic studies of xylose reductase from *Pichia stipitis*. Appl Microbiol Biotechnol.

[B46] Petschacher B, Leitgeb S, Kavanagh KL, Wilson DK, Nidetzky B (2005). The coenzyme specificity of *Candida tenuis *xylose reductase (AKR2B5) explored by site-directed mutagenesis and X-ray crystallography. Biochem J.

[B47] Kostrzynska M, Sopher CR, Lee H (1998). Mutational analysis of the role of the conserved lysine-270 in the *Pichia stipitis *xylose reductase. FEMS Microbiol Lett.

